# Design and implementation of an optimal laser pulse front tilting scheme for ultrafast electron diffraction in reflection geometry with high temporal resolution

**DOI:** 10.1063/1.4991483

**Published:** 2017-06-29

**Authors:** Francesco Pennacchio, Giovanni M. Vanacore, Giulia F. Mancini, Malte Oppermann, Rajeswari Jayaraman, Pietro Musumeci, Peter Baum, Fabrizio Carbone

**Affiliations:** 1 Laboratory for Ultrafast Microscopy and Electron Scattering, École Polytechnique Fédérale de Lausanne, CH-1015 Lausanne, Switzerland; 2 JILA, University of Colorado, 440 UCB, Boulder, Colorado 80309-0440, USA; 3 Laboratory of Ultrafast Spectroscopy, École Polytechnique Fédérale de Lausanne, CH-1015 Lausanne, Switzerland; 4 Particle Beam Physics Laboratory, Department of Physics and Astronomy, UCLA, Los Angeles, California 90095, USA; 5 Ludwig-Maximilians-Universität München, Am Coulombwall 1, 85748 Garching, Germany

## Abstract

Ultrafast electron diffraction is a powerful technique to investigate out-of-equilibrium atomic dynamics in solids with high temporal resolution. When diffraction is performed in reflection geometry, the main limitation is the mismatch in group velocity between the overlapping pump light and the electron probe pulses, which affects the overall temporal resolution of the experiment. A solution already available in the literature involved pulse front tilt of the pump beam at the sample, providing a sub-picosecond time resolution. However, in the reported optical scheme, the tilted pulse is characterized by a temporal chirp of about 1 ps at 1 mm away from the centre of the beam, which limits the investigation of surface dynamics in large crystals. In this paper, we propose an optimal tilting scheme designed for a radio-frequency-compressed ultrafast electron diffraction setup working in reflection geometry with 30 keV electron pulses containing up to 10^5^ electrons/pulse. To characterize our scheme, we performed optical cross-correlation measurements, obtaining an average temporal width of the tilted pulse lower than 250 fs. The calibration of the electron-laser temporal overlap was obtained by monitoring the spatial profile of the electron beam when interacting with the plasma optically induced at the apex of a copper needle (plasma lensing effect). Finally, we report the first time-resolved results obtained on graphite, where the electron-phonon coupling dynamics is observed, showing an overall temporal resolution in the sub-500 fs regime. The successful implementation of this configuration opens the way to directly probe structural dynamics of low-dimensional systems in the sub-picosecond regime, with pulsed electrons.

## INTRODUCTION

**FIG. 1.**
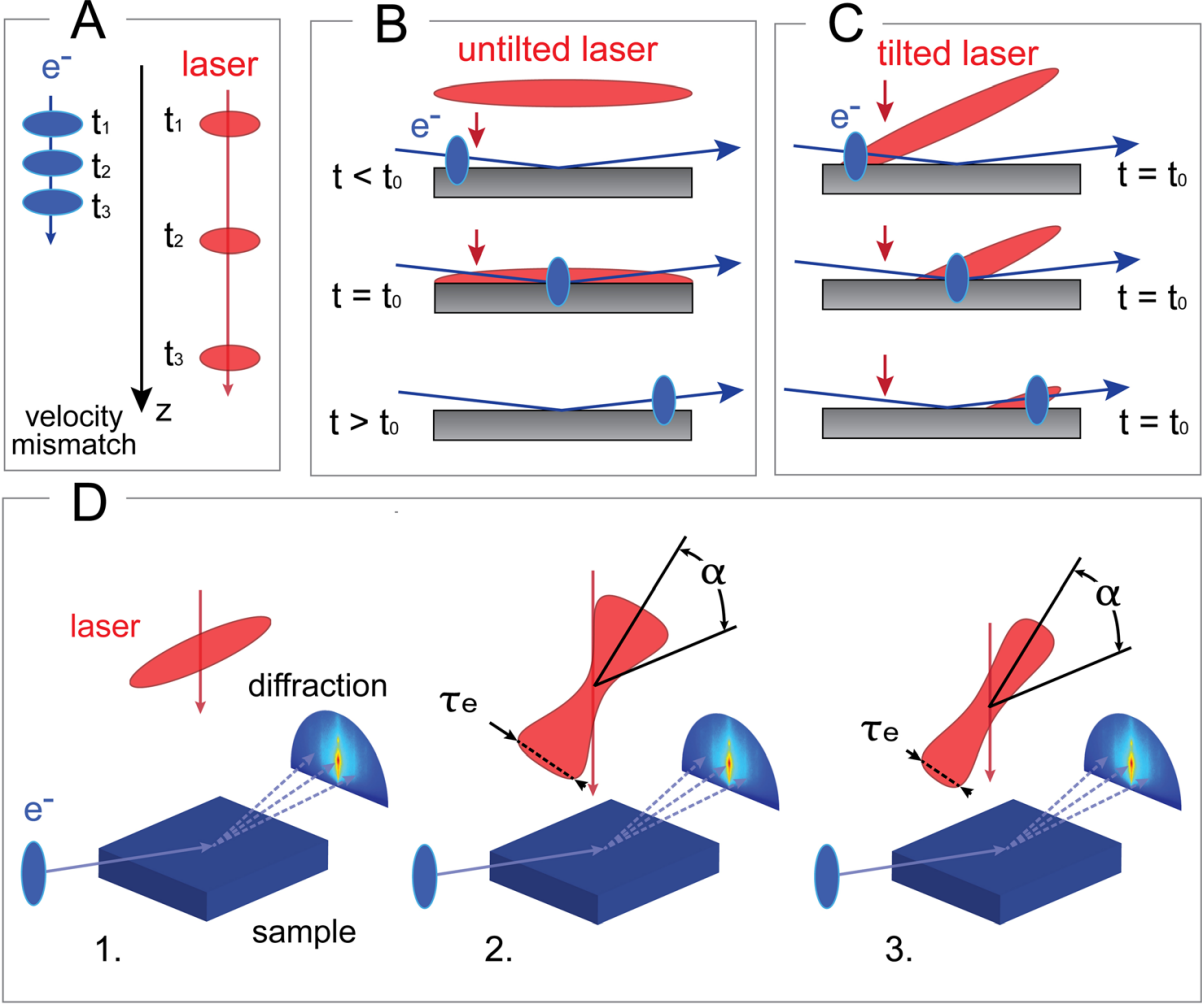
Group Velocity Mismatch (GVM) and tilting principles. (a) Representation of the GVM between the optical pump pulse (red) and the 30 keV electron probe (blue). During diffraction experiments in reflection geometry, the mismatch in group velocity causes the electrons to probe at different times/moments regions of the sample surface, which are excited simultaneously by the pump pulse (b). This can be avoided with a proper tilt of the wavefront of the pump pulse (c). (d) Comparison amongst three pumping geometries for a 30 kV UED setup. (d-1) An untilted laser pulse. (d-2) The tilted laser pulse (α = 71.9°) previously reported in Ref. 20, whose geometry leads to a temporal distortion τ_e_ of the pulse. (d-3) Tilted laser pulse (α = 70.7°) developed in LUMES UED setup to achieve a lower temporal distortion τ_e_.

The study of out-of-equilibrium behaviour in solids plays a fundamental role in the understanding of their functional properties, which are mediated by the ultrafast dynamics of their electronic and atomic structures.^1^ Ultrafast Electron Diffraction (UED), which exhibits a temporal resolution of hundreds of femtoseconds and a spatial resolution down to the atomic scale, is a powerful technique for dynamical structural investigations and has been used in a variety of systems and configurations.^2–4^ For example, it allowed to unveil the intermediate structures involved in many transitional phenomena in condensed matter, such as phase transitions,^5–9^ electron-lattice coupling,^10,11^ and anisotropic lattice excitation.^12^ More recently, it has been successfully used to investigate and characterize low dimensional nanoscale systems, unveiling order-disorder interplay in organic-inorganic 2D supracrystals,^13^ anisotropic expansion in carbon nanotubes,^14^ energy transport and dissipation in nanostructures,^15–17^ and rippling dynamics of free-standing graphene,^18^ and to probe bulk and surface heating mechanisms.^19^ Despite the demonstrated versatility of this technique, a major limitation remains the achievable time resolution, which is in part due to the different physical nature between the pump and the probe pulses. In a typical UED experiment, a light pulse initiates the dynamics of interest, which is then probed by an electron pulse. At 30 keV, the electrons speed is approximately 1/3 of the speed of light *c* [Fig. 1(a)]. In transmission geometry, with the pump and probe beam impinging almost collinearly on the sample surface and with a sample thickness generally below 100 nm, the mismatch in group velocities between electrons and light is negligible because of the narrow spatial interaction volume between the two pulses at the sample. On the contrary, in Ultrafast Reflection High-Energy Electron Diffraction (U-RHEED), the pump beam reaches the sample perpendicularly while the electron beam arrives at a grazing angle with respect to the surface. This causes the electrons to probe at different times/moments regions of the sample surface, which are excited simultaneously by the pump pulse. Thus, a mismatch in group velocities affects the overall time resolution [Fig. 1(b)]. As shown by the authors in Refs. 20 and 21, the time resolution can be improved by employing a pump pulse that is tilted by an angle α between the intensity front and the propagation direction. An optimal tilting angle allows the pump and the probe to hit the sample simultaneously all along its surface, as sketched in Fig. 1(c), improving the time resolution of the experiment. The proper front tilt *α* depends on both light and electrons velocities and can be calculated as reported in Ref. 20,

$$\alpha =\frac{\pi }{2}-\text{arctan}\left(\frac{v_{el}{\text{cos}\left(\beta \right)}^{-1}\text{sin}(\gamma \;-\beta )}{c-v_{el}{\text{cos}\left(\beta \right)}^{-1}\text{cos}(\gamma \;-\beta )}\right),$$ (1)

where *α* is the front tilt angle, *c* is the speed of light in vacuum, *v_el_* is the velocity of the electrons, *β* is the sample tilt, and *γ* is the incidence angle between the pump pulse and the sample surface. Considering *v*_el_ = 0.33⋅*c*, *γ *= 90°, and *β*  =  3°, the resulting tilting angle is *α* = 71.4°.

As a femtosecond laser pulse exhibits a substantial spectral bandwidth, a dispersive element and an imaging system can be combined to achieve a properly tilted wavefront.^22–24^ The tilting angle is linked to the angular dispersion by the relation $\alpha =\text{arctan}\left(\psi \lambda_{0}\right)$, where $\psi =M\frac{{\partial \theta }_{out}}{\partial \lambda }{|}_{\lambda_{0}}$. Here, *M* represents the magnification factor of the imaging element, *θ_out_* is the angle between the normal to the surface of the grating and the outgoing direction, and *λ_0_* is the central wavelength of the spectral bandwidth. The subsequent spatial separation of the different wavelengths in the spectrum is compensated by the imaging element. However, it has been demonstrated that dispersive elements such as gratings induce substantial temporal chirp in short pulses.^23^ This temporal chirp should in principle be compensated by the imaging element, but it has been observed^25,26^ that this is only true for the centre of the tilted beam. At a lateral distance Δ*x* from the centre of the pulse, which has a size of typically a few mm related to the required sample size in grazing-incidence diffraction experiments, the grating-to-lens distance is increased or reduced by a factor Δ*z* = Δ*x*⋅tan(*θ_out_*). At a distance Δ*z*, the pulse acquires the following duration:^23,26^

$$\tau \left(\Delta z\right)\approx \sqrt{1+\frac{{\left(2ln2\right)}^{2}{\Delta z}^{2}\psi^{4}\lambda_{0}^{6}}{\pi^{2}c^{4}\tau_{0}^{4}}\;},$$ (2)

where *τ_0_* is the Fourier-limited duration of the laser pulse before the dispersive element.

**FIG. 2.**
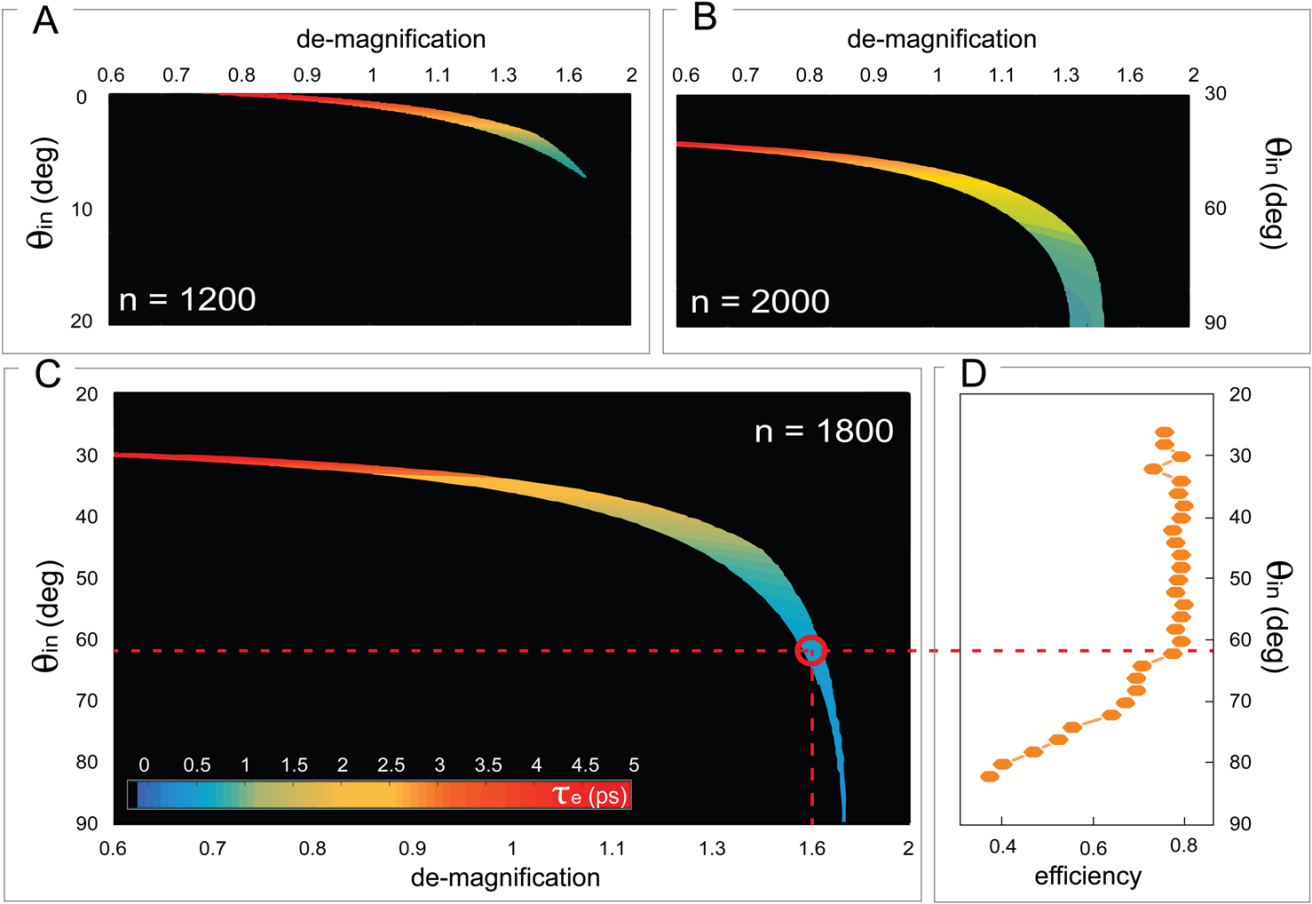
Parameters optimization in the design of an optimal tilting scheme. The temporal expansion of the tilted beam at 1.25 mm from its centre is reported as a function of the entrance angle *θ_in_* and the de-magnification factor *M*, for three gratings with *n* = 1200 [panel (a)], 1800 [panel (c)], and 2000 [panel (b)] groove/mm. The black areas define the set of parameters that do not provide a tilting angle α = 71.4° ± 1° and a beam dimension fitting to the geometrical constraints of the experimental chamber. Because the grating with *n* = 1800 groove/mm provides the lowest temporal chirp of the tilted beam, we monitored its efficiency as a function of θ_in_ and used it as additional constraint [panel (d)]. The parameters providing the best trade-off between temporal chirp and efficiency are defined by the red circle.

Consequently, a tilted pulse with tilting angle *α* = 71.9°, if produced at a grating configuration that is close to Littrow's conditions (*θ_in_* = *θ_out_* ≈ 53° on the grating), acquires a time chirp of around 1 ps at already 1 mm distance from the centre of the beam, as demonstrated in Ref. 26. This mechanism is sketched in Fig. 1(d-2). Such a temporal profile of the pulses is inadequate for grazing-incidence experiments on larger crystals with femtosecond time resolution. In Ref. 26, the authors solved the problem by using as outgoing angle from the grating a value of *θ_out_* = 0°. In this way, the grating surface is parallel to the objective plane of the imaging element, which can now properly compensate for the chirp all along its entire longitudinal dimension. Nevertheless, the achieved tilting angle in the initial demonstration was *α* = 61° and thus not optimal for the application in a UED setup using 30 keV electrons. Moreover, the higher deviation from Littrow's conditions on the grating surface is used, the less efficiency, defined as $\eta =\frac{P_{out}}{P_{in}}$ (where *P_in_* and *P_out_* are the incoming and outgoing beam powers) is achieved, providing not enough power to photoexcite the sample dynamics.

**FIG. 3.**
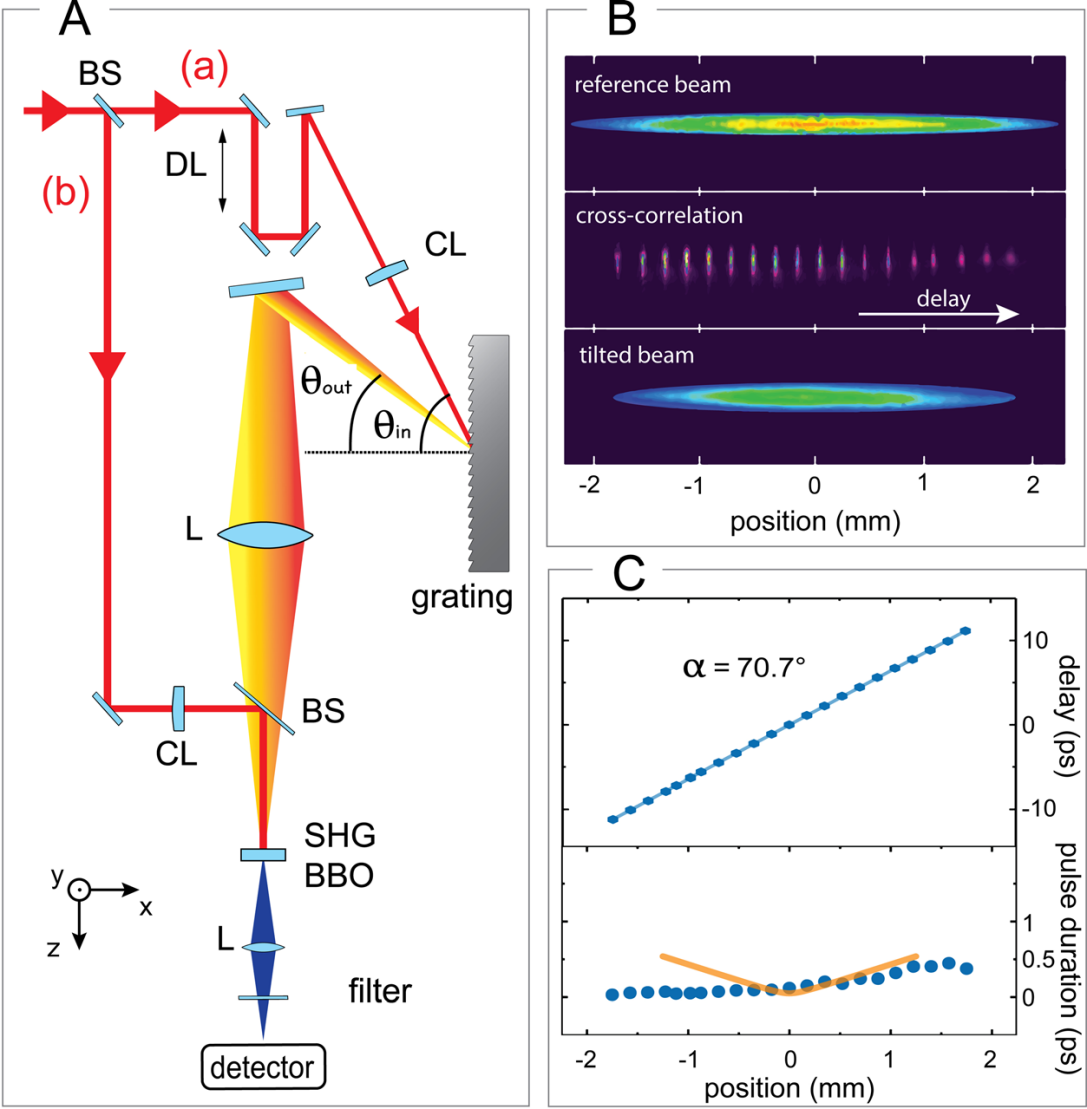
Optical characterization of the tilted laser pulse with λ_0_ = 800 nm. (a) Experimental setup to cross-correlate the tilted and an untilted pulse, as performed in Ref. 26. BS represents a 50:50 beam splitter, CL cylindrical lens, DL the delay line, and SHG BBO the nonlinear crystal responsible for the generation of the second harmonic, λ = 400 nm. The two angles used in our tilting setup are θ_in_ = 61° and θ_out_ = 35°. The y-axis indicates the out-of-plane direction. (b) Pictures of the 400 nm contributions taken by the camera: the second harmonic of the untilted beam (upper), of the tilted beam (lower) and their cross-correlation measured at different delays (middle). (c) Pulse-front tilt (upper) and duration (lower) of the pump beam from measured data (blue circles) and the calculation (orange solid line).

In this paper, we propose a modified tilting scheme that is able to deliver femtosecond light pulses with a tilting angle of 70.7° and an overall temporal width of better than 250 fs [see Fig. 1(d-3)]. This setup allows femtosecond resolution in U-RHEED experiments on mm-sized crystals. Its effectiveness has been proved by means of two characterizations, an optical cross-correlation measurement and a direct electron-laser temporal coincidence characterization based on a plasma lensing effect.^27,28^ Finally, a stroboscopic laser-pump/electron-probe study of a Highly Oriented Pyrolytic Graphite (HOPG) sample is reported and observed to produce femtosecond resolution.

## TILTING SCHEME

The light source in our experiment is a KMLabs Wyvern Ti:sapphire amplified laser, delivering 50 fs pulses with a pulse energy of 700 *μ*J at a central wavelength of λ_0_ ≈ 800 nm with a bandwidth of Δλ ≈ 40 nm and a repetition rate of 20 kHz.

Many parameters influence a tilting scheme for U-RHEED applications, such as the already mentioned *θ_in_* and *θ_out_* at the grating surface, its spacing parameter, the consequent angular dispersion, and the magnification factor due to the imaging element. In addition, there are also other parameters to take into account, such as its efficiency and the mechanical limitations related to the experimental setup. In our case, a lens-to-sample distance compatible with our vacuum system and the dimension of the dispersed beam at the entrance of the vacuum chamber to fit through the window.

**FIG. 4.**
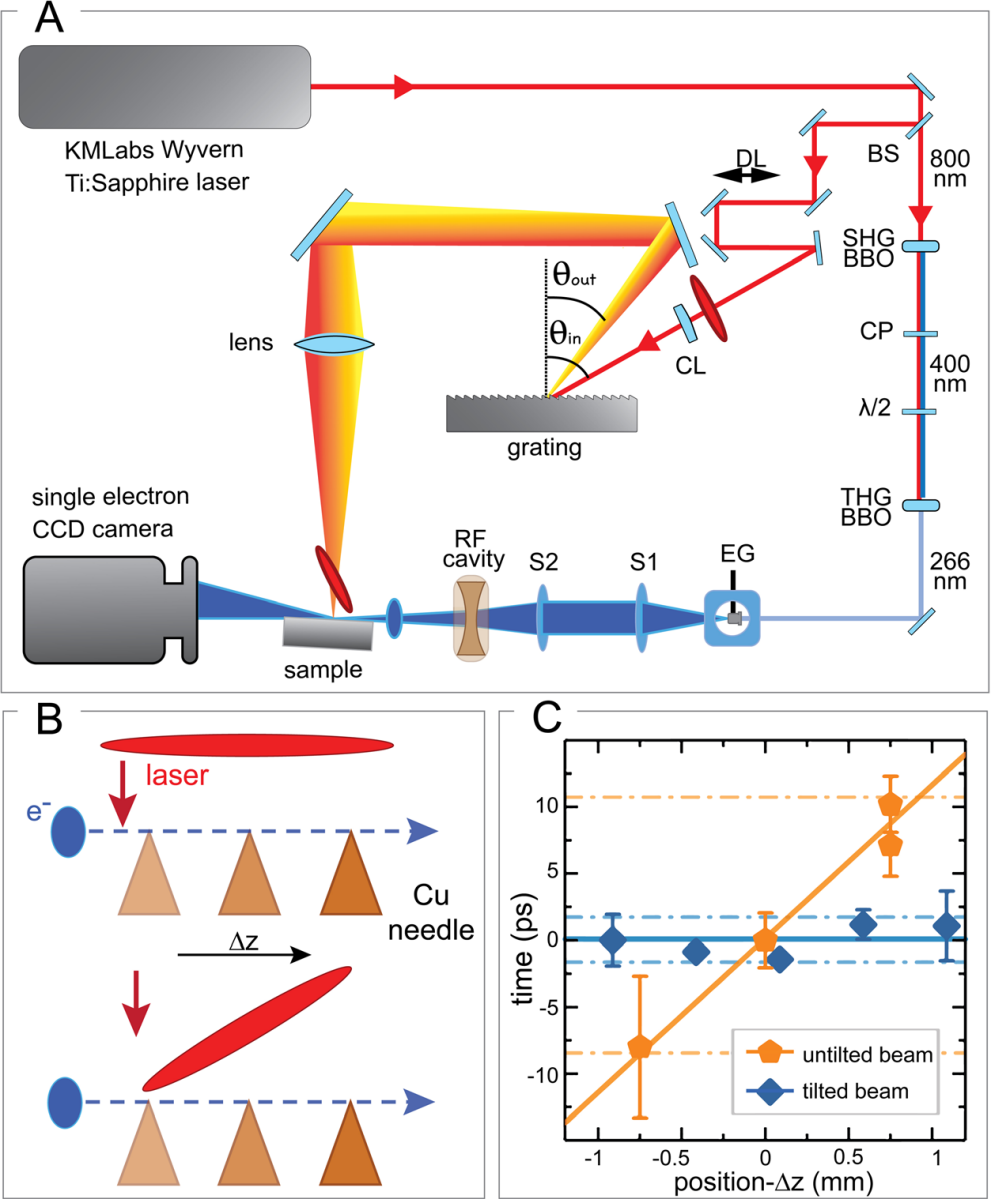
U-RHEED setup with implemented tilting system and in-chamber tilting characterization. (a) Schematic representation of the UED setup working in the reflection mode coupled to the tilting scheme described in the text. BS represents a 90:10 beam splitter, DL the delay line, CL a cylindrical lens, SHG and THG BBO the nonlinear crystals responsible for second and third harmonic generation, CP the group delay compensation plate, λ/2 the dual wave-plate, EG the electron gun, S1 and S2 a collimating and a focusing electronic lens, RF cavity our radio-frequency cavity operating in the TM_010_ mode to enhance the duration of the electron pulse. The two angles used in our tilting setup are θ_in_ = 61° and θ_out_ = 35°, defined as the exit angle of the central wavelength of our pulse. The ultra high vacuum system is omitted. Further details concerning the beamline parameters can be found in Ref. 29. (b) Schematics of the “plasma lensing effect” experiment as also performed in Ref. 20. A strong laser pulse is directed on a copper needle and thus generates a transient and localized plasma, which interacts with the propagating electron bunch inducing a sizeable distortion of its spatial profile. This lensing effect allows to define the time zero, t_0_, for a given position of the needle, which is then moved along the electron propagation direction. The results of this experiment for the two cases of not tilted (orange) and tilted (blue) pump beam are shown in (c), where the position of t_0_ is plotted as a function of the needle position.

To design an optimal tilting system while taking into account the high amount of variables and constraints involved, we performed an iterative calculation. Three gratings with different spacing parameters (*n* = 1200, 1800, and 2000 groove/mm) were evaluated. We considered all the possible combinations of *θ_in_* and magnification *M*, while keeping as constraints the desired tilting angle *α* = 71.4° ±1°, the lens-to-sample distance *i* > 25 cm, and the longitudinal dimension of the beam at the chamber window. For each combination of the parameters, the temporal chirp at 1.25 mm from the pulse center was computed, and it is now reported in Figs. 2(a)–2(c). Black areas in the maps correspond to conditions not satisfying the aforementioned constraints. As shown in the figure, only few combinations can provide a suitable tilting angle, and only the grating with *n* = 1800 groove/mm shows a sub-picosecond temporal chirp for the pulse. Once the suitable grating is selected, the power efficiency of the zero order diffraction was measured [see Fig. 2(d)] and adopted as an additional constraint. We have thus identified as the best trade-off between the achievable time chirp of the tilted pulse (sub-500 fs at 1.25 mm from the center of the beam) and the power efficiency of the system (η=0.8) the following parameters: an off-Littrow configuration with *θ_in_* = 61° and *θ_out_* = 35°, a grating-to-lens distance *p* = 52 cm, a lens-to-sample distance *i* = 32.5 cm, and thus a demagnification factor *M* = 1.6 [evidenced by the red circle in Fig. 2(c)]. During the optimization process, we only considered an imaging system with focal length *f* = 200 mm because of the geometrical constraints of the setup. A sketch of the final tilting scheme is presented in Figs. 3(a) and 4(a).

## OPTICAL CHARACTERIZATION

The obtained tilted pulse has been optically characterized by adopting the cross-correlation method demonstrated in Ref. 26. As presented in Fig. 3(a), a 50:50 beam splitter divides the collimated beam coming from the light source in two portions: one is used to generate the tilted beam (a) and the other is used as a reference beam (b). The first portion is directed through the cylindrical lens and afterwards on the grating to generate the tilted beam, according to the scheme explained above. It is delayed with respect to the non-tilted reference beam by means of a retroreflector mounted on a movable linear stage. A *β*-barium-borate (BBO) crystal with a thickness of 200 *μ*m is positioned at the lens-to-sample distance in our experiment, *i* = 32.5 cm. The imaging lens (*f* = 200 mm) generates a 3.5 × 0.2 mm^2^ spot size at the BBO surface.

The non-tilted reference beam is directed through a cylindrical lens providing a linear focus with dimension 4.5 × 0.2 mm^2^ on the BBO surface. It reaches the BBO coming from slightly above with an angle of 2°–3° (y-z plane) with respect to the tilted beam [see Fig. 3(a)].

As the 800-nm pulses interact with the BBO crystal, two individual contributions at 400 nm are generated, corresponding to the second harmonic of both the reference and the tilted beams [Fig. 3(b), above and below, respectively]. The residual 800-nm light is filtered out by the use of a chromatic filter. When spatiotemporal overlap is achieved on the nonlinear crystal, a third contribution is generated, representing the cross-correlation of the two incoming beams. At a given time delay, only a small region of the tilted pulse is temporally overlapped with the reference beam. Consequently, the spatial profile of the cross-correlation signal corresponds to a narrow stripe. The non-collinearity of the two incoming beams allows the cross-correlation signal to not overlap spatially with the individual contributions, which are removed after the crystal making use of a tunable slit.

A progressive delay scan provides a left-to-right shift of the cross-correlation signal. The cross-correlation signal obtained at different delays is shown in Fig. 3(b), middle panel. The position and width of this signal during the scan provide information on the tilt angle and on the pulse duration at a defined longitudinal position, respectively.^26^ Figure 3(c) (above) shows the measured pulse front tilt that we observed to be a straight line with an angle of 70.7° with respect to the horizontal. Figure 3(c) (below) contains the measured tilted pulse duration (blue circles) as a function of the distance from the centre of the beam toward the edges, as compared to the calculated pulse duration (orange solid line). Despite the small deviation from the ideal tilting angle (71.4°), a substantial agreement between experimental data and calculation is observed, confirming also that the temporal chirp of the tilted pulse is well compensated and it is smaller than 250 fs (≈the time resolution of our electron probe^29^) for more than 80% of the tilted pulse longitudinal dimension. The mismatch between the data and the computation is imputed to the model implemented, which was not taking into account the depth of focus of the Gaussian beam, and its asymmetry to a slight deviation of the grating-to-lens distance to the optimal one.

## U-RHEED SET-UP AND CHARACTERIZATIONS WITH ELECTRONS

The tilting optics have been integrated into our UED set-up [see Fig. 4(a)]. A 90:10 beam splitter is used to separate the 800-nm fundamental beam into two portions. The first represents the pump line, where the tilting scheme described above has been implemented, while the second portion is used to generate a 266-nm beam through a third harmonic generation process in nonlinear crystals. This UV pulse back-illuminates the Ag:sapphire photocathode of our Electron Gun (EG) and thus generates the electron bunches which are accelerated by a 30-kV potential. Two solenoids are used to correct the spatial divergence of the electrons, while a radio-frequency (RF) compression cavity^30^ working in the TM_010_ mode and synchronized with the laser oscillator compensates for the temporal divergence of the beam while maintaining 10^4^–10^5^ electrons per pulse. After the interaction with the sample, the electrons are detected by a Single-Electron Charge-Coupled Device (CCD). A detailed description of our UED setup can be found in Ref. 29.

To characterize the tilting at the sample position *in situ* in the UED setup, we adopted the method demonstrated by the authors in Ref. 20. A copper needle (apex size = 100 *μ*m) is placed on the sample holder such that it crosses the path of the electrons. With the proper magnetic lens configuration, a shadow image of the needle is obtained on the CCD camera. When hit by the pump pulse, a transient and localized plasma is generated on the needle surface by multiphoton ionization^27^ and distorts the spatial profile of the incoming electron beam (“plasma lensing effect”^28^) The relative change in each image with respect to a reference image taken before the excitation allows us to determine the time-zero, *t_0_*, defined as the temporal overlap between the pump and the probe pulses, with a precision of ∼1 ps.^20,28^ During the experiment, the position of the needle is varied along the propagation direction of the electron beam, and the value of *t_0_* is measured for each spatial coordinate. We performed the experiment with both the tilted pump pulse and an untilted reference pulse. As schematically shown in Fig. 4(b) (above) and derived from the experimental data in Fig. 4(c) (orange pentagons), when using the untilted pump the electron pulse overlaps at different times for different positions of the needle, showing an expected dispersion of ∼10 ps/mm. Instead, while using the tilted beam configuration, the *t_0_* remains constant for any position of the needle in the measured range of ∼2 mm [see schematics in Fig. 4(b) (below) and blue diamonds in Fig. 4(c)].

## TIME-RESOLVED EXPERIMENT ON HOPG

The overall time resolution provided in reflection geometry by our new tilting method was tested on a system of current interest: graphite. In graphite, Near IR light is known to excite a subset of phonon modes often referred to as Strongly Coupled Optical Phonons (SCOPs).^11^ These modes are primarily high-energy optical phonons^31^ which later decay via anharmonic couplings into all other lattice vibrations.

The mechanism for their population was first inferred via ultrafast terahertz spectroscopy^32^ and photoemission,^33^ clocking their characteristic thermalization time within 500 fs. The subsequent anharmonic decay was measured to occur with a time constant of 6–7 ps. The initial electron-SCOPs coupling in graphite is among the fastest global structural reactions ever measured in a solid, similar to the initial dynamics of VO_2_.^5,34^ However, observation of SCOPs excitation times so far always came from indirect methods that were probing the electronic structure alone. A hint of ultrafast structural rearrangement was obtained in Ref. 12 by monitoring the temporal evolution of the Debye-Waller effect of a single crystal of graphite at different fluences. A weak kink in the decay of a certain Bragg peak intensity was observed, but precise interpretation was obstructed by the too low time resolution of that experiment (around 700 fs).

In what follows, we show that our improved pulse-front tilting scheme in combination with the radiofrequency pulse compression technology in our reflection UED set-up allows for the direct structural observation of the characteristic time-scale of 500 fs associated with the population of the SCOPs, in excellent agreement with the photoemission results. In the present study, we use a low excitation fluence (6.5 mJ/cm^2^) to prevent any surface charge dynamics to influence our results.^12,35–37^

**FIG. 5.**
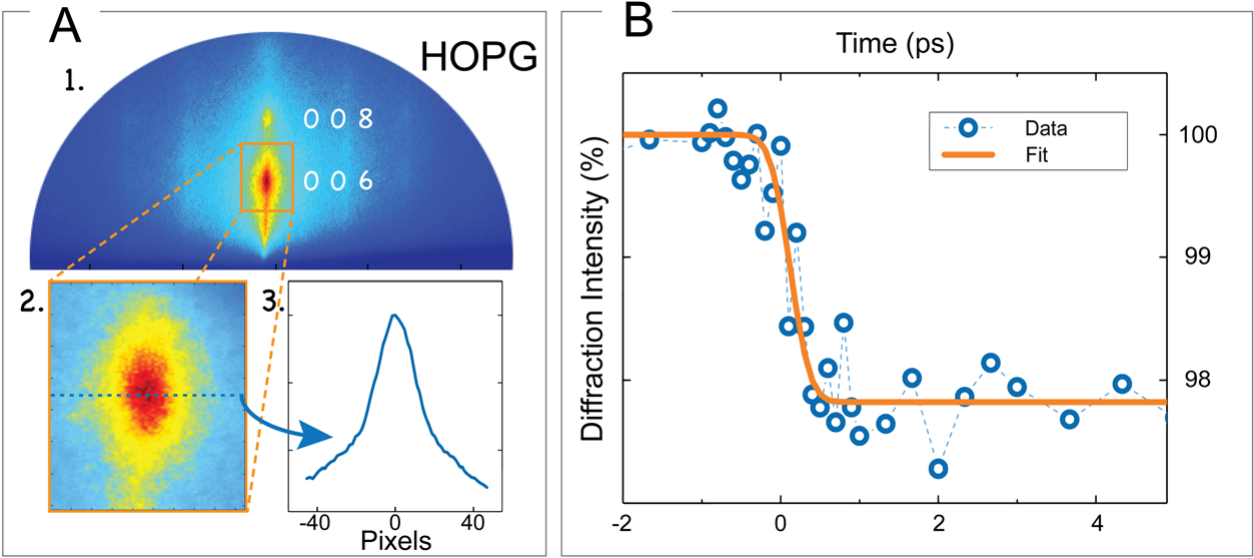
(a) Time-resolved reflection high-energy electron diffraction (RHEED) experiment in highly oriented pyrolytic graphite (HOPG): (a-1) representative diffraction pattern with labeled diffraction orders. (a-2) Details of the diffraction spot corresponding to the (006) plane family, whose integrated intensity profile (a-3) is analyzed. (b) Diffraction intensity evolution of the (006) Bragg spot with time. The observed dynamics corresponds to energy transfer from excited electrons to strongly coupled-optical-phonons (SCOPs), having a time constant τ* *= 500 fs.

We studied an “AGraphZ” HOPG sample from Optigraph GmbH, whose mosaic spread is 0.4° ± 0.1°. A static diffraction pattern is recorded [Fig. 5(a-1)], where the Bragg peaks corresponding to the (006) and (008) plane families are distinctly identified.

In the time-resolved experiments, a non-magnetic stainless-steel shield with a central hole is used to prevent the pump beam scattered light from reaching the CCD detector, thus reducing the background noise level. However, only a smaller portion of the diffraction pattern can be imaged on the CCD. For the time-resolved experiments, we selected the (006) reflection—shown in Fig. 5(a-2) as obtained before *t_0_*. Its profile is shown in Fig. 5(a-3). After optimizing the spatial overlap of the electron beam and laser spot at the sample surface, we monitored the intensity variation of the (006) Bragg peak as a function of the delay time between the pump and the probe. The results are plotted in Fig. 5(b), as blue open circles, while the orange solid line corresponds to the best least-squares error function fit of the data. The observed intensity decay evolves with the expected time constant *τ* ≈ 500 fs. Such a time-scale can be observed in the temporal evolution of the (006) Bragg reflection because the impulsive population of SCOPs is known to trigger strong c-axis dynamics.^12,38–43^

It is worth noting that although the light-induced population of SCOPs in graphite has already been observed in transmission geometry,^11^ we point out that this study provides the first direct measurement of the short time constant *τ* with U-RHEED on a 3D sample surface, which is not nanostructured and therefore more ideally resembles crystalline graphite than ultrathin flakes. The overall ultrafast structural dynamics, ps response, and longer time thermalization in graphite have already been investigated in detail in the literature.^11,12,31–47^

## CONCLUSIONS

In conclusion, by means of an iterative calculation approach, we have been able to identify and develop an optimal optical tilting scheme for ultrafast electron diffraction working in reflection geometry with 30 keV electrons. The multiple experimental characterizations presented in the paper demonstrated the validity of our method, which allowed us to achieve an overall temporal resolution in the sub-500 fs when using 10^4^–10^5^ electrons per pulse in a reflection diffraction scheme. The successful implementation of this configuration, which exhibits surface sensitivity with atomic-scale space and sub-picosecond time resolution, opens the path to the direct observation of the structural dynamics in solids, surfaces, and low-dimensional nanoscale systems such as quantum dots or two-dimensional materials.
